# A fully automated pipeline for mining abdominal aortic aneurysm using image segmentation

**DOI:** 10.1038/s41598-019-50251-8

**Published:** 2019-09-24

**Authors:** Fabien Lareyre, Cédric Adam, Marion Carrier, Carine Dommerc, Claude Mialhe, Juliette Raffort

**Affiliations:** 1 Cardiovascular Surgery Unit, Cardio Thoracic Centre of Monaco, Monaco, Monaco; 2 Université Côte d’Azur, University Hospital of Nice, Nice, France; 3 Laboratory of Applied Mathematics and Computer Science (MICS), CentraleSupélec, Université Paris-Saclay, Paris, France

**Keywords:** Anatomy, Cardiology, Diseases, Aneurysm

## Abstract

Imaging software have become critical tools in the diagnosis and the treatment of abdominal aortic aneurysms (AAA). The aim of this study was to develop a fully automated software system to enable a fast and robust detection of the vascular system and the AAA. The software was designed from a dataset of injected CT-scans images obtained from 40 patients with AAA. Pre-processing steps were performed to reduce the noise of the images using image filters. The border propagation based method was used to localize the aortic lumen. An online error detection was implemented to correct errors due to the propagation in anatomic structures with similar pixel value located close to the aorta. A morphological snake was used to segment 2D or 3D regions. The software allowed an automatic detection of the aortic lumen and the AAA characteristics including the presence of thrombus and calcifications. 2D and 3D reconstructions visualization were available to ease evaluation of both algorithm precision and AAA properties. By enabling a fast and automated detailed analysis of the anatomic characteristics of the AAA, this software could be useful in clinical practice and research and be applied in a large dataset of patients.

## Introduction

Abdominal aortic aneurysm (AAA) is associated with high rates of morbidity and mortality^1^. The only curative treatment available relies on surgical approaches including open and endovascular surgery^2^. Recent advances in medical imaging technology have led to the development of medical image analysis software allowing to create reconstructions of the AAA. Such software is useful to measure aortic and vessels lengths and diameters in order to optimize the sizing of endografts^2^. Nevertheless, commercialized software currently available for CT-scan images are semi-automatic and require human intervention to initiate aorta localization and measurements of vessels. In addition, they are not constructed to provide automatic quantitative analysis of AAA anatomic characteristics such as vessel calcifications or the presence of intra-luminal thrombus. Automatic segmentation of the AAA is challenging due to the heterogeneity of the AAA morphology and the low discrimination between the AAA and the surrounding tissues^3^. Automatic software would be of interest for clinical practice to facilitate the sizing for surgeons and standardize the procedure. In addition, it would be useful for clinical research to provide a fast and detailed analysis of the anatomic characteristics of the AAA.

In this paper, we present our pipeline and describe the key algorithms that we implemented to develop a fully automated methodology, independent of human manual appreciation, in a training dataset of CT-scan images of AAA. This software allows an automatic detection of the main characteristics of AAA: the aneurysmal localization in the aorta, the distance to the renal and iliac arteries, the presence of calcifications and intraluminal thrombus.

## Materials and Methods

### Dataset and method

CT-scans images were obtained from 40 patients with an infrarenal AAA who had multidetector CT scanners with arterial-phase intravenous injection of contrast liquid. The protocol was approved by the University Hospital of Nice Review Board. All methods were carried out in accordance with the French Regulatory Health Authorities and informed consent was obtained from all subjects. Images were given in Digital Imaging and Communications in Medicine (DICOM) format providing a matrix of size 512 × 512 for each slice with a pixel size of 0.81 +− 0.13 mm and a slice thickness of 0.90 +− 0.35 mm. Scans came from 10 institutions and 8 different models of 4 manufacturers (GE Medical Systems, Philips, Toshiba and Hitachi Medical Corporation). Six scans showed arterial systems with existing stent.

**Figure 1:**
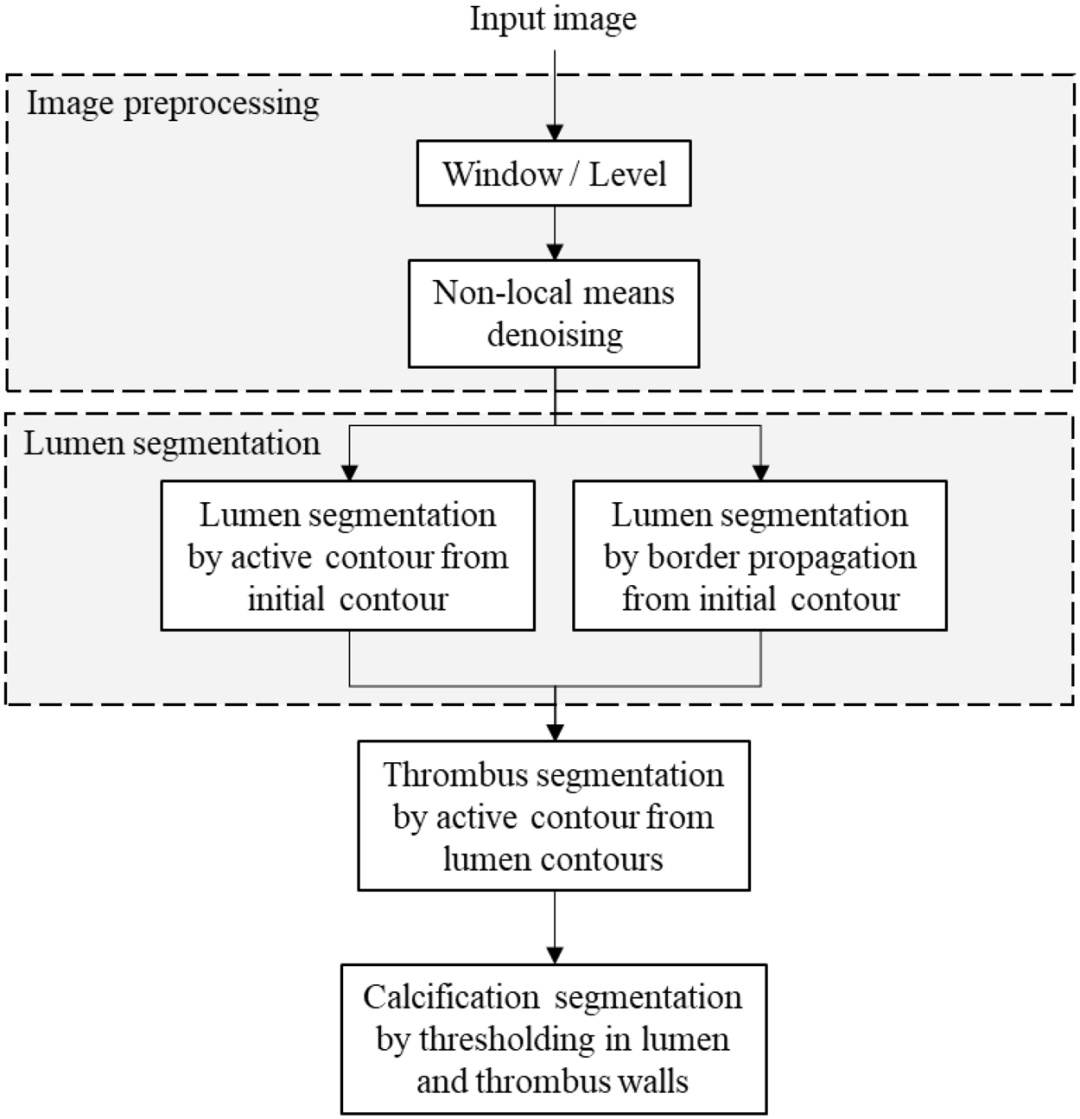
Flowchart of the proposed automatic segmentation method.

The method used for aneurysm segmentation from CT-scan images consists in four sequential steps: image preprocessing, segmentation of the aortic lumen, segmentation of the aortic thrombus, and segmentation of the aortic calcifications. The pipeline of the proposed automatic segmentation method is presented in Fig. 1.

### Image preprocessing

The preprocessing is a mandatory step before image segmentation. Standard image filtering and denoising algorithms for medical imaging were used: Gaussian, median and bilateral filters and fast non-local means denoiser from the OpenCV library^4^. Grayscale pixel intensities *G* are computed from the Hounsfield values *H* using the following formula:

$$G=\,{\rm{\min }}(255,\,\max (0,(H-(L-\frac{W}{2}))\ast \frac{255}{W})),$$

where *L* is the window level and *W* is the window width.

In this study, a window level of 40 and a window width of 400 are used to obtain an adapted contrast to lumen segmentation. For the thrombus segmentation, the window width may be reduced to 300 or 200 to increase the contrast.

### Lumen segmentation

**Figure 2:**
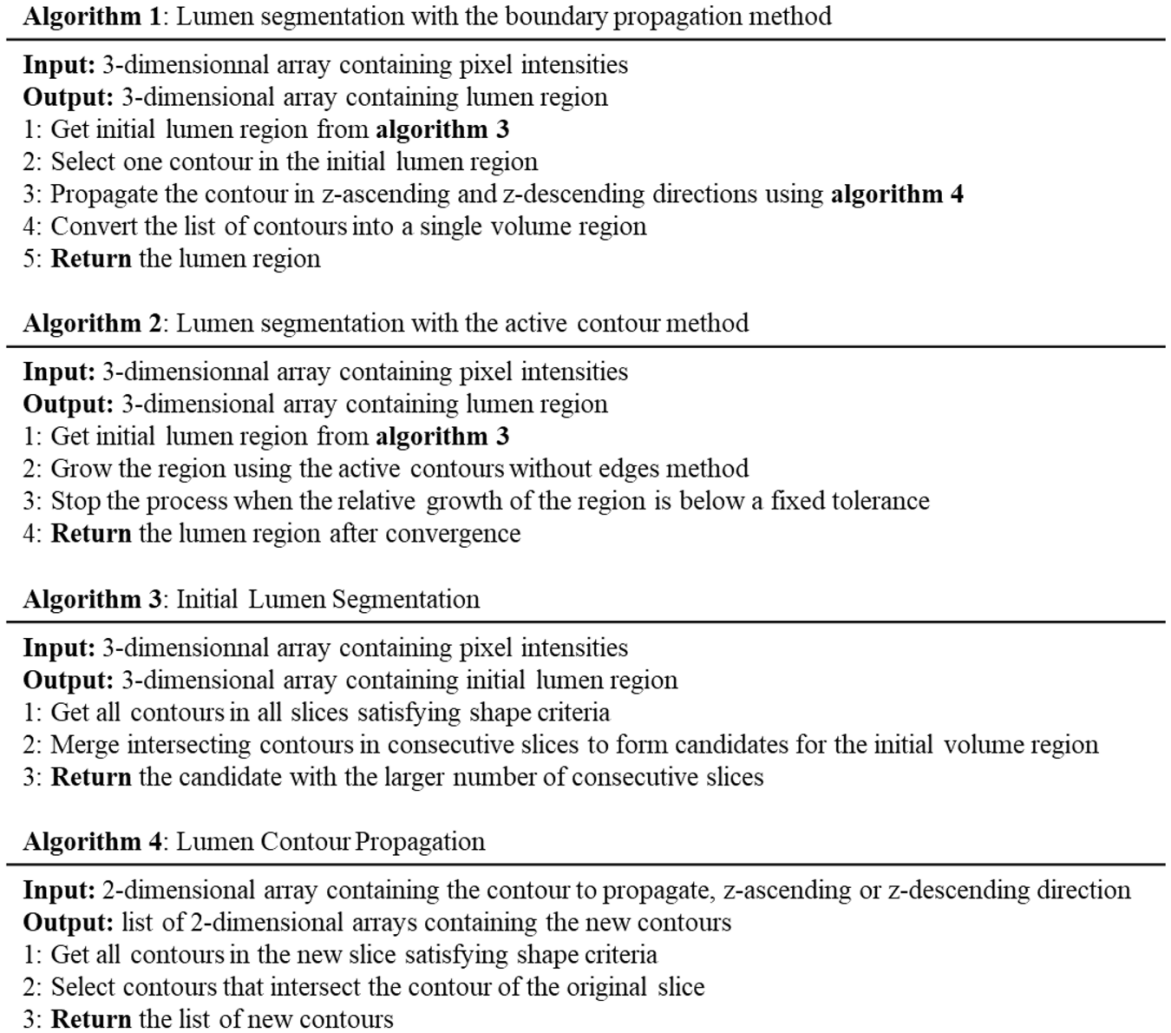
Pseudo-codes used of the lumen segmentation.

The methodology for lumen segmentation was designed to allow an automatic detection and localization of the aorta. The method was built to discriminate contrasted arterial system from surrounding tissues which have pixel gray scale close to the agent contrast such as the spine and the vertebrae. The pseudocodes of the lumen segmentation algorithms are given in Fig. 2.

### Lumen segmentation with the boundary propagation method

**Figure 3:**
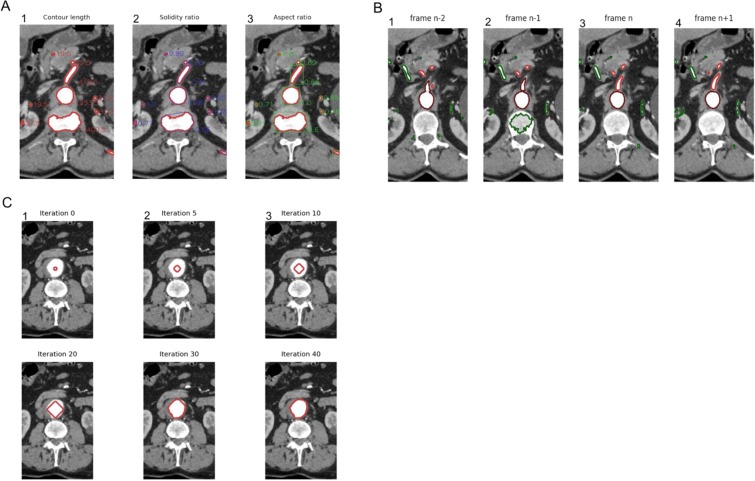
Segmentation of the aortic lumen. (**A**) Representative images of criteria used to discriminate the aortic lumen on CT-scan images. (1) Contour length, (2) Solidity ratio, (3) Aspect ratio. (**B**) Discrimination of an artery originating from the aorta. The artery (in light red) is detected by the intersection with the aorta (in dark red) from a pool of contours (green borders). (1) Frame n-2, (2) Frame n-1, (3) Frame n, (4) Frame n + 1. (**C**) Representative images of the propagation method from an initial contour (in red) over 40 iterations until the convergence is achieved.

The method allows to localize a section of the aorta and then segment arterial system by propagation from the aorta position. The algorithm detects the contours potentially belonging to the aorta lumen in each slice by thresholding the slice. A border following algorithm^5^ implemented in the OpenCV library is used to detect the candidates to arterial system lumen in the binary slice produced. Candidates set in each slice is pruned based on a priori shape properties of the aorta: size, aspect and solidity. The size of the contour is given by its area and contour length, as these properties of aorta have been studied deeply in medical research^6^ (Fig. 3A-1). The solidity of a contour represents its smoothness, which is expected to be high for blood vessels compared to bones tissues (Fig. 3A-2). The aspect of a contour provides an indication of its roundness, which is expected to be high for the aorta in horizontal slices of the body^7^ (Fig. 3A-3).

The aspect *A* properties of a contour are given by:

$$A=\frac{\min (w,h)}{\max (w,h)},\,S=\frac{a}{ha},$$

where *w* and and *ℎ* are respectively the width and height of the minimum area rotated rectangle enclosing the contour and *ℎa* are respectively the area of the contour and the area of the convex hull of the contour.

Candidates that exhibit best oriented-ellipse fitting properties, with high aspect and solidity values, are kept^7^.

Contours are pulled together as they intersect themselves following the body vertical direction. The aorta is localized as the largest set of contours pulled together.

From the section of aorta, the arterial system is detected by propagation through intersection of contours in a set of contours. This larger set of contours is obtained with the same border following algorithm as the aorta initial localization but with less severe shape properties constraints. A result is shown on a stack of 4 frames in Fig. 3B.

Spine and lumen regions may have similar pixel values. Moreover, these two structures are close and may intersect in some frames. This can lead to a loss of accuracy in the automatic lumen segmentation, with a propagation of the arterial system detection in the spine region. To increase the robustness of the method, spine region is segmented in a first step, and removed from the area of research for the lumen segmentation. The border propagation method presented is used to segment the spine, and blacked out for the lumen segmentation.

### Lumen segmentation with the active contour method

In this paper, several initial lumen surface candidates are computed in each image using a threshold-based contour detection. Each aorta volume candidate is computed propagating an initial contour in z-direction. Connected components are then extracted and compared to automatically detect the starting lumen region. The length in z-direction is selected as a relevant metric for the comparison. This process is automatic and thus does not require the end-user to manually select the lumen initial region.

Active contours methods are used to segment 2D and 3D regions. In our approach, we consider the active contours without edges algorithm since it does not rely on well-defined borders. We minimize the ACWE functional $F({c}_{1},{c}_{2},{\mathscr{S}})$ of a surface ${\mathscr{S}}$ Given by:

$$F=\mu \ast {\mathscr{A}}({\mathscr{S}})+\nu \ast {\mathscr{V}}(inside({\mathscr{S}}))+{\lambda }_{1}\mathop{\int }\limits_{inside({\mathscr{S}})}|{\rm{I}}({\rm{x}})-{c}_{1}|dx+{\lambda }_{2}\mathop{\int }\limits_{inside({\mathscr{S}})}|{\rm{I}}({\rm{x}})-{c}_{2}|dx,$$

where the nonnegative parameters *μ*, *v*, *λ*_1_ and *λ*_2_ control the strength of each term, ${\mathscr{A}}$ is the area operator, *V* is the volume operator, *I*(*x*) is the intensity of a pixel *x* and *c*_1_ and *c*_2_ are given by:

$${c}_{1}=\frac{{\int }_{outside({\mathscr{S}})}{\rm{I}}({\rm{x}})dx}{{\int }_{outside({\mathscr{S}})}dx},\,{c}_{2}=\frac{{\int }_{outside({\mathscr{S}})}{\rm{I}}({\rm{x}})dx}{{\int }_{outside({\mathscr{S}})}dx}.$$

Figure 3C illustrates the evolution of an initial contour, in red, over 40 iterations until convergence is achieved.

The convergence is based on the ratio between volume increase at each iteration and the total volume of the region. We use the following stopping criterion:

$$\frac{d{\mathscr{V}}}{{\mathscr{V}}} < tol,$$

where $d{\mathscr{V}}$ is the variation of the volume between two successive iterations, ${\mathscr{V}}$ is the volume of the region and $tol$ is a user defined tolerance set to 10^−6^.

In this study, a morphological smoothing based on binary dilate and erode operators is applied at each iteration. In the two-dimensional case, the binary erosion E and dilation *D* are compiled using the following formula:

$$E(x(i,j))=\mathop{\min }\limits_{(\delta i,\delta j)\in P}x(i+\delta i,i+\delta j),\,D(x(i,j))=\mathop{\max }\limits_{(\delta i,\delta j)\in P}x(i+\delta i,i+\delta j),$$

where where *P* is a pattern given in^8^, *x*(*i*, *j*) is the binary value of a pixel located at coordinates *(i, j)*. In practice, to balance the contribution of both operators, we alternate the two following smoothing operators *S*_1_ and *S*_2_ through iterations:

$${S}_{1}(x(i,j))={\rm{E}}\,\circ \,{\rm{D}}(x(i,j)),\,{S}_{2}(x(i,j))={\rm{D}}\,\circ \,{\rm{E}}(x(i,j)).$$

The dependency of the algorithm results to its parameters values were evaluated through the Dice Similarity Coefficient (DSC) of the segmented volumes^9^:

$$DSC=2\frac{|{R}_{1}\cap {R}_{2}|}{|{R}_{1}|+|{R}_{2}|},$$

where *R*_1_ and *R*_2_ are the segmented regions. When there is no overlap, the outcome of DSC is 0, and for complete overlap the outcome is 1.

### Thrombus segmentation

In our segmentation pipeline, the computation of the lumen region is an important step for the thrombus segmentation. The lumen region is set as initial level set for segmenting the thrombus region. A fully automated segmentation of the thrombus represents a technical challenge. Semi-automated approaches require that the end-user pick a region of the thrombus to calibrate the intensity of the pixels in this region. In this paper, the thrombus intensity is automatically computed using the initial thrombus segmentation such that an end-user intervention is not required. As for the segmentation of the lumen, a morphological snake ACWE is applied to grow the thrombus region. However, a different LUT filter is applied to increase the contrast of the CT images. Since the contrast is increased, a than twenty-five iterations are required to segment the thrombus region. The assumption that the Gaussian filter is also applied to reduce the noise of the image. Practically, less thrombus region has a globally ellipsoidal shape in 2D which is of major importance for the fully-automated procedure. A morphological smoothing based on dilate and erode operators is applied at each iteration but with a higher strength than for the lumen case. Instead of using 3 × 3 patterns^8^, 5 × 5 and 7 × 7 patterns are applied. This results in a smoother contour in each slice of the CT volume.

### Segmentation of calcifications

**Figure 4:**
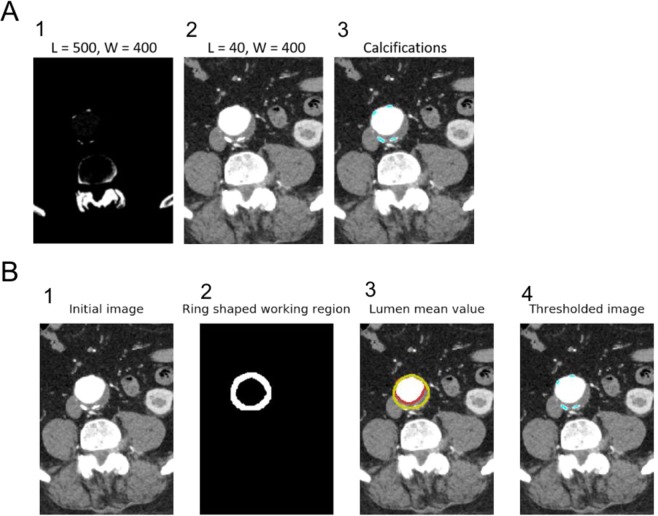
Segmentation of calcifications. (**A**) Visualization of calcified tissues and contrast medium in CT-scan images using LUT algorithm. L corresponds to grey level; W corresponds to the window (range of values of Hounsfield units converted into grey levels). (**B**) Steps to discriminate calcifications in the aortic lumen and the intraluminal thrombus.

The algorithm developed was initially created based on injected CT-scan images but must work in non-contrasted images. It relies on a previous detection of the wall of the aorta as presented. The LUT and level sets commonly used to visualize arterial system in injected CT-scans unable the proper visualization of calcifications but calcifications have significantly higher absorption of rays than contrasted blood, therefore a higher Hounsfield unit (Fig. 4A).

Each slice is analyzed within a segmentation mask, obtained from the contour of the aorta wall. A morphological dilate operator is applied to the aorta mask, and a morphological erode of the same size, which are then subtracted to the original mask. The working region is therefore reduced to a ring-shaped region to search for calcifications.

Finally, a threshold is applied in the ring-shaped working region. All the volume elements, or voxels, with Hounsfield units above the threshold are considered as calcifications. The threshold is computed from the mean of the inner lumen pixel values multiplied by a constant factor superior to one. An illustration of these steps is provided in Fig. 4B.

### Visualization

2D visualization is performed using a pixel matrix with a gray-scale color map.

It allows to render slices of CTA scans in z-direction one-by-one. 2D visualization allows to evaluate the contours of the segmented regions and to compare with manually drawn contours by experts. Metrics can then be applied to benchmark the proposed methods quantitatively.

We also use 3D visualization to render, in real time, 3D complex surfaces such as the arterial system. Numerical results can be quickly and qualitatively examined by a medical end user.

### Validation methodology

The method was compared with respect to evaluation of ground truth provided by a human expert. A quantitative validation of our algorithm was performed using CT-scan image data sets acquired from 40 patients with infrarenal AAA. For each patient, a manual segmentation of the aortic lumen and the thrombus was independently performed by an expert vascular surgeon. The manual tracing was performed using a contour drawing tool of the image processing software Image J (version 1.52). The segmentation was sequentially performed by analyzing slices of the infrarenal aorta on a mean length of 152 +/− 54 slices per patient.

The segmentation results were evaluated using the following distinct metrics:

1. Overlap based metrics.
  - the Dice Similarity Coefficient (DSC) of the segmented volumes^9^.
  - the Jaccard index (JAC), defined as:
  $${\rm{JAC}}={\rm{TP}}/({\rm{TP}}+{\rm{FP}}+{\rm{FN}})$$
  JAC is larger than DSC except at the extrema {0, 1} where they are equal. The two metrics are related according to the formula:
  $${\rm{JAC}}={\rm{DSC}}/(2-{\rm{DSC}})$$
  - the True Positive Rate, or Sensitivity, measures the portion of positive voxels in the ground truth that are also identified as positive by the segmentation being evaluated^9^.
  $${\rm{Sensitivity}}={\rm{TP}}/({\rm{TP}}+{\rm{FN}})$$
  where TP are the true positive and FN the false negative
  - The True Negative Rate, or Specificity, measures the portion of negative voxels (background) in the ground truth segmentation that are also identified as negative by the segmentation being evaluated^9^.
  $${\rm{S}}{\rm{p}}{\rm{e}}{\rm{c}}{\rm{i}}{\rm{f}}{\rm{i}}{\rm{c}}{\rm{i}}{\rm{t}}{\rm{y}}={\rm{T}}{\rm{N}}/({\rm{T}}{\rm{N}}+{\rm{F}}{\rm{P}})$$
  where TN are the true negative and FP the false positive
2. Volume based metric.
  The volumetric similarity (VS) is a measure that considers the volumes of the segmented areas. It represents the absolute volume difference divided by the sum of the compared volumes^9^.
  $${\rm{VS}}={\rm{1}}-{\rm{abs}}({\rm{FN}}-{\rm{FP}})/({\rm{2}}\ast {\rm{TP}}+{\rm{FP}}+{\rm{FN}})$$
3. Surface distance based metric.

The Hausdorff distance is the maximum distance of a segmented volume to the nearest point in the other segmented volume^10^.

$${\rm{HD}}({\rm{A}},\,{\rm{B}})=\,{\rm{\max }}({\rm{h}}({\rm{A}},\,{\rm{B}}),\,{\rm{h}}({\rm{B}},\,{\rm{A}}))$$

where h(A, B) is called the directed Hausdorff distance and given by the formula:

$${\rm{h}}({\rm{A}},{\rm{B}})=\,{\rm{\max }}\,{\rm{\min }}|{\rm{a}}-{\rm{b}}|$$

$${\rm{a}}\in {\rm{A}}\,{\rm{b}}\in {\rm{B}}$$

where |a − b | is the Euclidean distance.

## Results

### Lumen segmentation with the boundary propagation method

**Figure 5:**
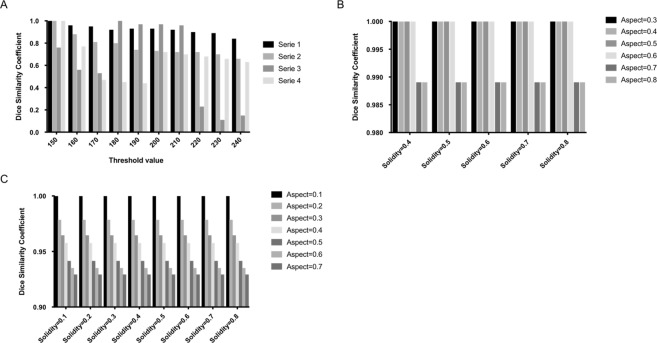
Lumen segmentation with the boundary propagation method. (**A**) Sensitivity of found arteria volume to threshold. Series 1 and 2 represent “ideal” scans, when the aorta is well contrasted, surrounded by soft tissue and without stent graft. Serie 3 represents scans with stent graft or low contrast product. Serie 4 represents scans where the aorta is surrounded by other hard tissues. (**B**) Dice Similarity Coefficient of found arterial system volume to the restrictive morphological parameters. (**C**) Dice Similarity Coefficient of found arterial system volume to permissive morphological parameters.

The dependency to the value of the threshold converting the grayscale image in a level set is low in case of “ideal” scans: when the aorta is surrounded by soft tissues, without stent, well contrasted (see series 1 and 2 in Fig. 5A). In these cases, increasing the threshold decreases slowly the depth of the arterial system detected. However, if the threshold value is high, detection may be “stopped” by stent grafts reflection, too small arteries or improperly applied contrast liquid (false negative). Segmentation results were evaluated using the Dice Similarity Coefficient (DSC). The DSC of series 3 (Fig. 5A) illustrates cases where stent graft or low contrast product “stops” the detection of the lumen if the threshold is high. On the opposite, the algorithm can allow propagation in other tissues touching the aorta (false positive) if the threshold value is low (see series 4 in Fig. 5A). A medium value of 200 enables to robustly detect a large part of the arterial system with few false positive. In the 40 test cases, 6 cases have 1 to 3 slices with false positives and 6 cases include false negatives parts (aorta portion or a renal arteria departure undetected). Two of the 6 false negative cases are due to metallic interference of stent grafts.

Two set of physiological parameters values are used: restrictive values to be sure to exclude false positive while trying to locate the aorta, then permissive values to propagate in the entire vascular system. The algorithm result is more dependent of permissive parameters than to restrictive set (see Fig. 5B,C). However, the dependency to those parameters values remains very low compared to the threshold.

### Lumen segmentation with the active contour method

**Figure 6:**
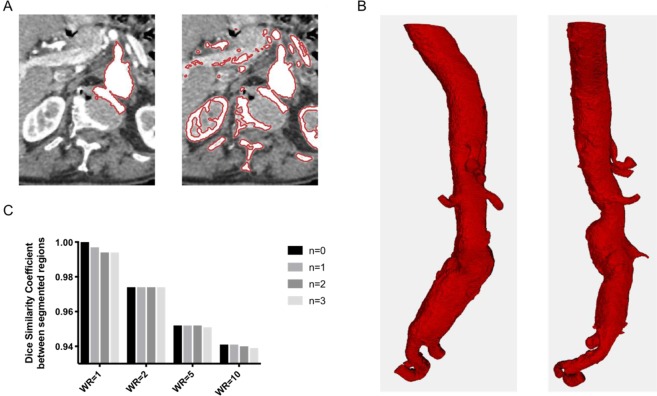
Lumen segmentation with the active contour method. (**A**) Intersection between spine and lumen regions in a slice using a ACWE method without smoothing (left) and a threshold-based contour extraction (right). (**B**) 3D segmentation of lumen region after 50 iterations of morphological ACWE. (**C**) Dice Similarity Coefficient between segmented regions for different weight ratios (WR) and numbers of smooth operations (n).

A morphological snake ACWE is applied to segment 2D and 3D regions. Figure 6A illustrates the intersection between spine and lumen regions. Since both regions have similar pixel intensities, a threshold-based contour extraction or a ACWE method is inaccurate in this case. To segment the lumen region, a smoothing, i.e. a balloon force, is applied at each iteration of the morphological snake algorithm.

Practically, less than fifty iterations are required to segment the lumen region containing at least: the aorta, two renal and two iliac arteries. Figure 6B shows the 3D segmentation of lumen region after 50 iterations of morphological ACWE. Abdominal aorta, celiac trunk artery, superior mesenteric artery, iliac arteries and renal arteries are segmented. Internal and external iliac arteries are also visible. One hundred and fifty iterations were sufficient to properly discriminate the aorta and the arteries.

Figure 6C shows DSC for different weight ratios and numbers of smooth operations. The weight ratio is defined by the quotient of the inner weight parameter to the outer weight. The reference is the area segmentation without smoothing and with a weight ratio set to 1. Up to three smoothing operations are applied at each iteration of the ACWE algorithm. An increase of the weight ratio leads to a decrease of the DSC, up to 6% of the reference value. In this example, the DSC is slightly impacted by the number of smoothing operations. For a reasonably selective weight ratio of 5, the lumen segmentation error, defined by the DSC between the segmented region and the reference region, is bounded by 5%.

### Computational efficiency

The sequential version of the morphological snake method requires a significant computational time, which can be an issue when dealing with a lot of patient CTA-scans in clinical research, as well as in the case of decision making for a surgical emergency. However, a parallel version of the active contour technique has been developed in this work. The resulting parallel active contour require less than one minute to perform a segmentation using several cores on a standard laptop. The boundary propagation method is faster than the active contour since it is a threshold-based technique.

### Thrombus segmentation

**Figure 7:**
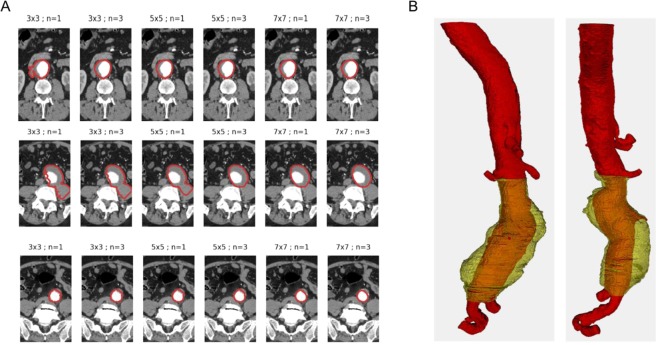
Thrombus segmentation. (**A**) Thrombus segmentation with different patterns (strength) and number of smooth operators applied at each iteration of the growing active region. (**B**) 3D segmentation of thrombus region located between the two iliac arteries and the two renal arteries.

Several patterns for the smoothing operator have been assessed when segmenting the thrombus: 3 × 3, 5 × 5 and 7 × 7 pattern. Qualitatively, they respectively correspond to weak, medium and strong smoothing. Figure 7A represents the results of the thrombus segmentation for three different CT-scan slices. Each row corresponds to a single slice. Three different patterns, or strength, are used and the number of smooth operators applied at each iteration of the ACWE algorithm varies from one to three. It highlights that, for this segmentation, a weak smoothing is not sufficient to properly segment the thrombus region since the morphological snake goes into the surrounding tissues. Three medium strength smoothing operations at each iteration or a single strong smoothing operation are sufficient to extrapolate the elliptic shape of the thrombus wall, without well-defined edges.

The combination of all these techniques have shown a high robustness and accuracy in the thrombus segmentation for 2D and 3D cases. The region grows and stops even with partially defined borders. This case occurs when neighboring organs or arteries are close to the thrombus. Figure 7B shows the result of the lumen and thrombus segmentations in a 3D case. The thrombus region is located between the two iliac arteries and the two renal arteries. For the segmentation of the thrombus region, the lumen area is set as the initial levelset. A 7 × 7 pattern for the discrete smoothing operator is applied in order to segment quite smooth regions.

### Segmentation of calcifications

**Figure 8:**
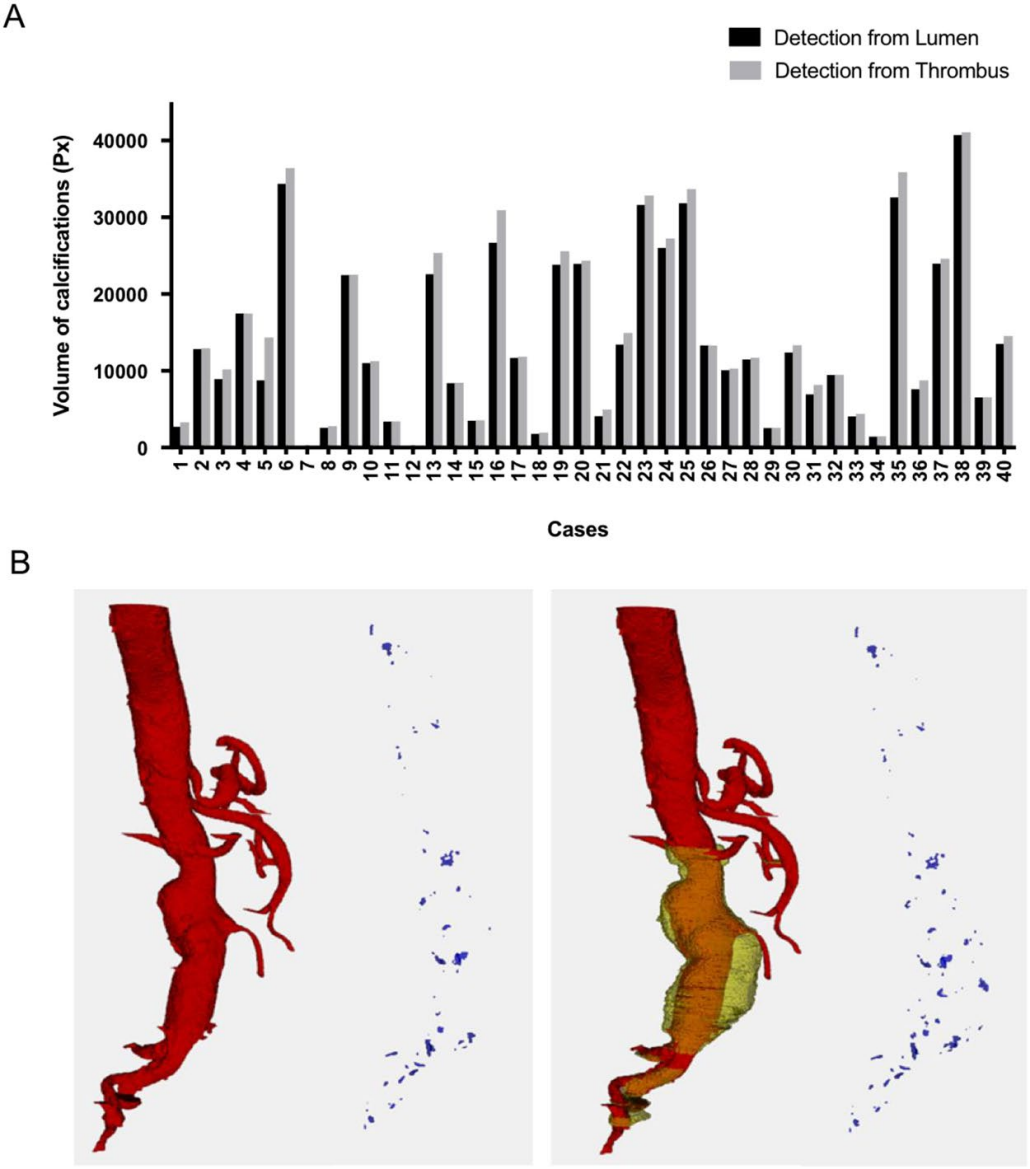
Segmentation of calcifications. (**A**) Volume of calcifications detected in CT-scan dataset. (**B**) Representative images of calcifications detected.

Calcifications are detected around arterial system wall, approximated by either the lumen or the thrombus contour. On the dataset, the use of the thrombus has led to an increase of the average volume of calcifications by 7.8% +− 0.11, as seen in Fig. 8A. Representative 3D images of calcifications detected along the aorta are shown in Fig. 8B.

### Validation of the methodology

**Figure 9:**
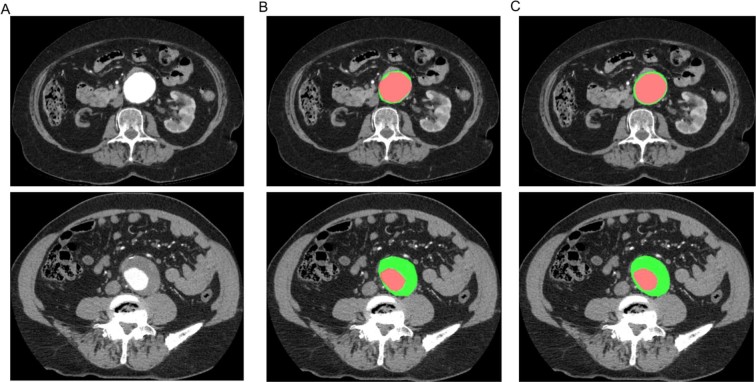
Representative images of the segmentation of the aortic lumen (in red) and the intraluminal thrombus (in green). (**A**) CT-scan cross section obtained from patients with infrarenal AAA. (**B**) Manual segmentation. (**C**) Automatic segmentation

**Figure 10:**
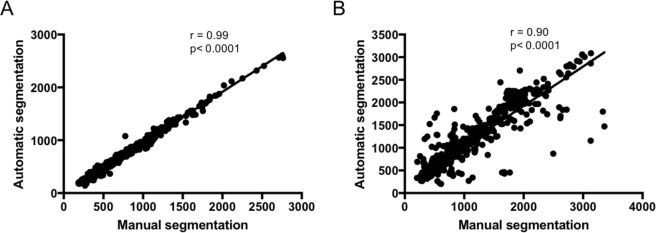
Correlations of the surfaces measured with manual segmentation by a human expert and automatic segmentation methods. (**A**) Segmentation of the aortic lumen (n = 620). (**B**) Segmentation of the thrombus (n = 525) r: Pearson’s coefficient correlation.

To evaluate the results of the segmentation obtained with our automatic pipeline, we performed a quantitative comparison with the results obtained from manual segmentation by an expert vascular surgeon on a dataset of 40 CT-scan from patients with infrarenal AAA. Representative images of the manual and the automatic segmentation of the aortic lumen and the intraluminal thrombus are presented in Fig. 9. For the aortic lumen, the analysis was performed on 620 slices and demonstrated an excellent correlation of the surfaces measured with the manual and the automatic segmentation methods, with a Pearson’s coefficient correlation of 0.99, P < 0.0001 (Fig. 10). The results on the metrics used to evaluate the segmentation errors are presented in Table 1. The mean volume similarity was 0.96 +/− 0.04; the mean sensitivity 0.90 +/− 0.06; the mean specificity 0.9997 +/− 0.0004; the mean Jaccard index 0.87 +/− 0.07; the mean Dice Similarity Coefficient 0.93 +/− 0.04 and the mean Hausdorff distance 1.78 +/− 0.38. For the segmentation of the thrombus, 525 slices were analyzed and the Pearson’s coefficient correlation between the 2 methods was 0.90, P < 0.0001 (Fig. 10). The mean volume similarity was 0.91 +/− 0.11; the mean Jaccard index 0.80 +/− 0.15; the mean Dice Similarity Coefficient 0.88 +/− 0.12 and the mean Hausdorff distance 2.13 +/− 0.61 (Table 2). For the automatic method, the segmentation time varied from 5 seconds to 1 minute per patient. For the human manual method, the segmentation time ranged from 25 minutes to 40 minutes per patient.

**Table 1:** Summarized results of the evaluation of the segmentation methodology for the aortic lumen on a data set of 40 CT-scan obtained from patients with infrarenal AAA.

Segmentation of the aortic lumen (620 slices analyzed)				
	Mean+/−SD	Min-Max	Median	Interquartile range
Surface from manual method (mm^2^)	652.8 +/− 404.3	185.5–2764.7	528.1	367.0–795.4
Surface from automatic method (mm^2^)	620.6 +/− 391.0	141.3–2604.4	503.1	351.9–760.7
Volume similarity	0.96 +/− 0.04	0.69–1	0.97	0.95–0.99
Sensitivity	0.90 +/− 0.06	0.45–0.99	0.92	0.88–0.95
Specificity	0.9997 +/− 0.0004	0.9924–1.0000	0.9997	0.9996–0.9999
Jaccard index	0.87 +/− 0.07	0.42–0.96	0.88	0.85–0.91
Dice Similarity Coefficient	0.93 +/− 0.04	0.59–0.98	0.94	0.92–0.96
Hausdorff distance	1.78 +/− 0.38	0.85–3.24	1.72	1.52–2.0

**Table 2:** Summarized results of the evaluation of the segmentation methodology for the thrombus on a data set of 40 CT-scan obtained from patients with infrarenal AAA.

Segmentation of the thrombus (525 slices analyzed)				
	Mean+/−SD	Min- Max	Median	Interquartile range
Surface from manual method (mm^2^)	1109.9 +/− 685.7	206.2–3359.1	924.8	538.8–1589.1
Surface from automatic method (mm^2^)	1149.0 +/− 665.3	202.2–3088.0	1034.5	556.5–1627.4
Volume similarity	0.91 +/− 0.11	0.40–1.00	0.95	0.90–0.98
Sensitivity	0.91 +/− 0.14	0.22–1.00	0.96	0.91–0.99
Specificity	0.9978 +/− 0.0029	0.9778–1.0000	0.9988	0.9974–0.9993
Jaccard index	0.80 +/− 0.15	0.12–0.96	0.85	0.78–0.89
Dice Similarity Coefficient	0.88 +/− 0.12	0.22–0.98	0.92	0.87–0.94
Hausdorff distance	2.13 +/− 0.68	0.88–6.48	1.98	1.69–2.39

## Discussion

The precision and robustness of the automatic detection method result from a complex combination of several techniques.

Image processing using filtering and denoising is essential for an accurate segmentation. Computer tomography machines generally produce an unwanted noise and decrease the image quality. The noise changes the pixel values and results in a heterogeneous image. Several authors aimed to compare different noise removal algorithms in medical imaging^11–13^. The authors used several measures to assess the quality of the denoising. They compared different filters, such as Wiener and median filters, and denoisers, such as wavelet approaches or non-local means. The latter approach requires much time compared to others. For this reason, other versions of non-local means have emerged, with the aim at being faster while keeping a satisfying accuracy. Noise removal for CT-scan imaging is still an active field of research^14,15^. Some investigators compared several filters and showed that a combination of median filter followed by Wiener filter is more effective to remove different noises present in CT images. In this study, the median filter exhibited the best compromise between image quality and computational efficiency.

Vascular segmentation is a challenging task and investigators proposed a large review of 3D vessel lumen segmentation techniques with models, features and extraction schemes^16–19^. Some authors compared several extraction schemes such as region growing approaches^20^ or active contours^21,22^. To cope with most complex applications, advanced techniques often rely on a combination of several models and features.

Boundary propagation and active contour methods are proposed in this paper to segment the lumen. Both methods exhibit a good accuracy for the aortic wall detection, from the celiac trunk artery, to the two iliac arteries. The main difference between 2D and 3D method results in the way the detection of the arterial system stops. Depending of the patient anatomy and the way the contrast liquid is applied, one method or the other will perform a more complete detection of the arteries. However, the accuracy of the process in regions with a metallic stent graft may be reduced due to the interferences with the CT machine. In the boundary propagation method, the lumen detection process may be artificially stopped in the graft zone due to the presence of high intensity pixels coming from foreign body interferences and decreasing the quality of the aortic wall. In this study, the combined use of boundary propagation method and active contour model demonstrated a high robustness, allowing to cope with the complex problem of automatically segmenting the lumen region from the spine for all patients.

Active contours methods are widely used to segment vascular regions. Two main approaches exist: the geodesic active contours (GAC)^23^ and the active contours without edges (ACWE)^24^. The ACWE does not rely on a preprocessed image, the method can handle not well defined borders. In both methods, a time-dependent partial differential equation (PDE) is solved, which is a computationally expensive operation. Marquez-Neila *et al*. have shown that some morphological operators, such as dilation and erosion, can be expressed as PDEs^8^. Moreover, they approximate the numerical solution of region growing PDEs by the successive composition of morphological operators. The morphological approach has several advantages over the PDE approach since the implementation is simpler, has fewer parameters, and no numerical instability issues. Besides, morphological algorithms are faster, which makes them suitable for processing large images and volumes such as those from CT scans. For robustness purpose, we have used a ACWE algorithm combined with several smoothing operations at each iteration. Larger numbers of smoothing operations lead to smoother segmentations. More recently, several approaches using deep learning techniques have been proposed^25–28^ to segment the lumen region. The approaches are based on deep convolutional networks that are able to extract deep feature: the first convolution layer extracts the low-level features, like edges, lines and corners, while the deeper the layers, the higher-level is the information they provide. The main limitation of CNN relies on the fact that this technique requires a large amount of training data due to the huge amount of parameters that it has. In this work, we have chosen to use a feature-based approach, which does not rely on a large amount of input data. A future work would be to generate synthetic data from the feature-based approach and use them in a deep neural network approach to speed up the segmentation process.

Spine segmentation using threshold-based methods and connected component extraction are essential to a robust and precise detection of the lumen.

The lumen segmentation using boundary propagation or active contour methods is used as the initial region for the thrombus segmentation. Thrombus and lumen segmentation approaches share similar techniques but, the edges of the thrombus are not so different from the surrounding tissues. Therefore, thrombus segmentation is a challenging problem since borders of thrombus are not well defined. Among popular methods applied to thrombus segmentation, one can cite active contours^29,30^, graph cut algorithms^31,32^ or non-parametric statistical gray-level appearance model-based^33^. Lalys *et al*. developed a thrombus segmentation method composed of an initial centerline detection with aortic lumen segmentation, an optimized pre-processing stage and the use of a 3D deformable model. This approach showed accurate results with respect to manual delineations with a Dice score of 0.86 +/− 0.06 for abdominal aorta sections on pre-operative CT-scans^34^. Recently, Lopez-Linares *et al*. have proposed a fully automatic detection and segmentation of abdominal aortic thrombus using a deep convolutional neural network^35^. The pipeline was trained, validated and tested in 13 CT-scan of patients with AAA. The comparison with manually delimited volume resulted in a Dice score of 0.82 +/− 0.07. In our study, the thrombus segmentation was achieved using active contour methods with a strong balloon force to extract the elliptic feature of the thrombus. Our results showed the agreement of our method with the ground truth defined by the experienced human, with a Dice score of 0.88 +/− 0.12. These results are within the same range of other previously published methods.

The segmentation of calcifications was performed using a threshold-based method in the aortic wall region. Several methods to detect calcifications in abdominal aorta have been proposed in the literature so far, most of them rely on a previous localization of the aorta walls and considers only high-density areas within the segmented volume. A few fully-automatic methods were proposed. Isgum *et al*. proposed a supervised learning algorithm for the segmentation of calcifications^36^. The main limitation of this type of methods relies on the requirement of a large amount of training data, 433 CT-scans have been manually annotated. In this study, we propose an automated calcification segmentation from contrasted scans and performed without learning datasets. In this objective, several threshold-based methods have been proposed in the literature: some investigators applied a fixed threshold in Hounsfield unit^37,38^ whereas others proposed a refined method, based on an adaptive thresholding in low contrasted scans^39^. Here we propose a similar approach, but with a detection in high contrasted datasets, enabling a fully automated evaluation of calcifications from segmented aorta wall in CT-scans. As shown in the results, segmenting the thrombus can lead to increase significantly the number of calcifications detected.

Comparing images to evaluate the quality of segmentation is an essential step to validate the methodology. Segmentation evaluation consists in comparing two segmentations methods by measuring the distance or similarity between them^9^. We compared our new automatic method to the ground truth of manual segmentation performed by an expert using several metrics including overlap, volume and surface distance based metrics. This work resulted in a proper segmentation of most patients of the dataset, despite some low contrast CT-scans or metallic interferences due to the presence of stent grafts. Our results demonstrated the agreement of the method with manual segmentation and the robustness of the pipeline to detect and automatically segment the lumen and the thrombus of AAA. It presents the advantage to reduce segmentation time and user interaction. An adaptation of the nominal value of some parameters remains available for a precise characterization of patients with specific anatomies. The expertise of a medical professional remains essential when interpreting the 2D and 3D visualizations to evaluate if a new set of parameter values is required or not.

Several methods have been previously proposed for aneurysm segmentation. De Bruijne *et al*. developed semi-automatic methods for aneurysm sac segmentation as well as thrombus segmentation inspired from the active shape model segmentation^40,41^. The methods demonstrated accurate segmentation but required minimal user interaction to initiate the detection of the aorta. Another semi-automatic method based on a two-step segmentation for the inner and for the outer aneurysm border has been proposed and compared to manual segmentation. While it exhibited relative errors close to those obtained with human experts, it still required a minimal user interaction^42^. In the method proposed by Zhuge *et al*., the user intervention is not required except to identify the most proximal and distal slices of the aneurysm^43^. The results on a data set of 20 CT-scan were compared to the gold standard established by manual tracing from experts. The mean volume overlap was 95.3% +/− 1.4 and the mean segmentation time per patient was reduced to 7.4 min +/− 3.8 vs 20 to 30 min per patient with the human manual method. Another semi-automatic method to segment the lumen interface and the aortic wall of AAA was developed based on graph cut theory^44^. The comparison of the results with those obtained by human tracing from experts based on three metrics including the maximum aortic diameter, the volume overlap and the Hausdorff distance demonstrated the reliability of the method. Finally, Joldes *et al*. recently proposed a finite element analysis-based approach to analyze the rupture potential of an AAA and presented the preliminary results from 48 cases^3^. The software system consists of a collection of programs to enable image segmentation, geometry creation, meshing, finite element analysis and rupture potential index computation. While the analysis is performed automatically, the segmentation of the AAA and the intraluminal thrombus remains semi-automatic.

Based on our results, several perspectives can be suggested. Further studies to compare the quantitative data obtained from this pipeline with established published scores on a larger dataset of CT-scan images would be of interest. The availability of the data is a key issue for training and validation of new imaging processing techniques. In practice, it is extremely difficult to obtain datasets from previously published cohorts as medical data sharing is subjected to legal and ethical restrictions and is not publically available^45^. The development of publically-available large-scale computational resources and datasets would be a step forward in the development of automated-imaging analysis.

The full-automation of this method offers interesting perspectives to easily and quickly characterize a high quantity of CT-scans of AAA, which could be useful for applications in clinical research. This method brings a standardized characterization of AAA and could be useful in clinical practice to homogenize and facilitate the sizing of endograft. Even if further studies are required to validate the method on a larger set of patients, this fully automated pipeline could bring new insights in imaging processing and could have potential applications in both clinical practice and clinical research.

## Data Availability

All data generated or analysed during this study are included in this published article.
